# The Role of Alpha-Dystrobrevin in Striated Muscle

**DOI:** 10.3390/ijms12031660

**Published:** 2011-03-04

**Authors:** Masayuki Nakamori, Masanori P. Takahashi

**Affiliations:** 1 Department of Neurology, Osaka University Graduate School of Medicine, 2-2, D-4, Yamadaoka, Suita, Osaka 565-0871, Japan; E-Mail: masayuki_nakamori@urmc.rochester.edu; 2 Department of Neurology, University of Rochester Medical Center, 601 Elmwood Avenue, Box 645 URMC, Rochester, NY 14642, USA

**Keywords:** dystrobrevin, syntrophin, dystrophin, DGC, muscular dystrophy, signaling, intermediate filament, splicing

## Abstract

Muscular dystrophies are a group of diseases that primarily affect striated muscle and are characterized by the progressive loss of muscle strength and integrity. Major forms of muscular dystrophies are caused by the abnormalities of the dystrophin glycoprotein complex (DGC) that plays crucial roles as a structural unit and scaffolds for signaling molecules at the sarcolemma. α-Dystrobrevin is a component of the DGC and directly associates with dystrophin. α-Dystrobrevin also binds to intermediate filaments as well as syntrophin, a modular adaptor protein thought to be involved in signaling. Although no muscular dystrophy has been associated within mutations of the α-dystrobrevin gene, emerging findings suggest potential significance of α-dystrobrevin in striated muscle. This review addresses the functional role of α-dystrobrevin in muscle as well as its possible implication for muscular dystrophy.

## 1. Introduction

α-Dystrobrevin is a member of the dystrophin gene family with homology to the cysteine-rich carboxy-terminal domain of dystrophin [1,2]. α-Dystrobrevin was identified as human and mouse homologues of an 87 kDa phosphoprotein found at the Torpedo electric organ post-synaptic membrane [1,3,4]. In striated muscle, dystrobrevin and dystrophin are both localized to the cytoplasmic face of the sarcolemma, and form a macromolecular complex with a variety of proteins and glycoproteins, termed dystrophin-glycoprotein complex (DGC) [5]. The DGC plays crucial roles in maintaining the structural integrity of muscle fibers by linking the extracellular matrix to the subsarcolemmal cytoskeleton, and also provides a scaffold for signaling molecules. The abnormalities of the DGC are recognized to be responsible for several forms of progressive muscular dystrophies. The absence of dystrophin results in loss of the entire DGC from the sarcolemma and leads to Duchenne muscular dystrophy (DMD) [6]. Mutations in sarcoglycan genes, one of the components of the DGC, result in several types of limb-girdle muscular dystrophy (sarcoglycan-deficient LGMD or SD-LGMD). Although α-dystrobrevin directly associates with dystrophin and sarcoglycan complex [7,8], no mutation has been identified in muscular dystrophy patients hitherto. However, α-dystrobrevin knockout mice, which demonstrate mild myopathy, have provided clues of its potential functions in striated muscle [9]. Recently, α-dystrobrevin is known to be subject to extensive alternative splicing [4,10], which results in changes in its subcellular distribution and function in muscle [11]. Furthermore, a number of binding partner of dystrobrevin have been identified. In this review, we focus on the recent findings regarding the alternative splicing of α-dystrobrevin, its interactions with other proteins, and implications for muscular dystrophy.

## 2. α-Dystrobrevin Gene and Transcripts

The mammalian dystrobrevin protein family is comprised of α- and β-dystrobrevin which are encoded by the *DTNA* and *DTNB* gene, respectively [4,12]. α-Dystrobrevin is expressed predominantly in muscle and brain whereas β-dystrobrevin is expressed in non-muscle tissues [3,4,12]. The human α-dystrobrevin gene consists of 23 coding exons [10].

**Figure 1.**
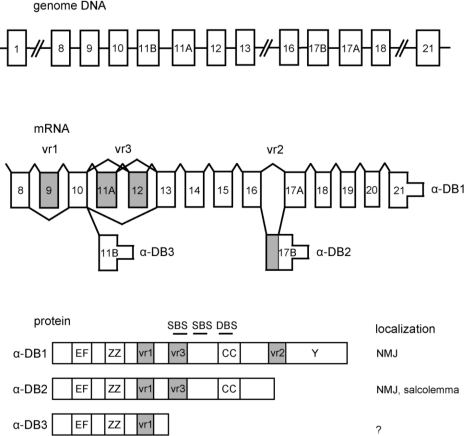
Alternative splicing of human α-dystrobrevin. Exons represented as thick boxes are translated segments and thin boxes indicate untranslated segments. The exon numbering is based on [10], which is different from that proposed by [18]. Introns and downstream flanking regions are represented by horizontal lines. Gray boxes represent the alternatively spliced exons: variable regions 1 (vr1), 2 (vr2), and 3 (vr3). The different isoforms of protein are represented below. The identifiable domains are boxed: EF, EF hand region; ZZ, zinc-binding domain; CC, coiled-coil domain; Y, unique tyrosine kinase substrate domain. Suggested syntrophin binding sites (SBS) and dystrophin binding site (DBS) are indicated. This figure is a modified version of an illustration in [17].

α-Dystrobrevin is known to be subject to extensive splicing regulation. The alternative usage of three exons—21, 17B, and 11B—generates transcripts of different lengths encoding three major α-dystrobrevin isoforms in human skeletal muscle: α-dystrobrevin 1, α-dystrobrevin 2, and α-dystrobrevin 3 [10] (Figure 1). Additional diversity is observed due to alternative splicing within the coding regions referred to as variable regions 1, 2 and 3 [4]. Firstly, variable region 1 (vr1) consists of a short exon encoding three amino acids (exon 9). In mouse, the transcripts including this exon are primarily restricted to brain [4,13], but are present in brain, heart, and skeletal muscle in human [10]. Variable region 2 (vr2) consists of exons 17A and 17B. Exon 17B encodes the unique *C*-terminal tail of α-dystrobrevin 2. The first 21 nucleotides of exon 17B are also found in the α-dystrobrevin 1 transcript as a result of splicing at a cryptic site [14]. In mouse, the expression of the vr2 region in the α-dystrobrevin 1 transcript appears to be developmentally regulated [13]. Lastly, variable region 3 (vr3) consists of exons 11A, 11B and 12. Exon 11B encodes the unique *C*-terminal tail of α-dystrobrevin 3. In α-dystrobrevin 1 and α-dystrobrevin 2, four in-frame alternatively spliced transcripts, may arise by joining exon 10 with either exon 11A, 12 or 13, or by joining exon 11A with either 12 or 13 (*i.e*., 10-11A-13, 10-12-13, 10-13, 10-11A-12-13) [10]. In mouse skeletal muscle, the splicing of vr3 has also been reported to be developmentally controlled [13,15,16]. Previous analyses of mouse myoblast cultures demonstrated a transition from α-dystrobrevin lacking vr3 (Δvr3) to α-dystrobrevin including vr3 (+vr3) during differentiation. We also showed that the frequency of vr3 inclusion increases in skeletal muscle tissue during normal postnatal development of mice [17]. However, we found that α-dystrobrevin Δvr3, which should be the fetal isoform in mouse, is expressed exclusively in adult human muscle, and the relative amount of α-dystrobrevin +vr3 is higher in human fetal, than in adult, cardiac muscle [17]. These results suggest a different, or possibly an opposite, mode of regulation of vr3 alternative splicing between human and mouse. Since this portion, as described below, corresponds to one of syntrophin binding sites, these results might imply a functional difference of α-dystrobrevin between mice and human. Recently, Bohm *et al.* extensively analyzed the differences in α-dystrobrevin splicing between human and mouse [18]. They also showed the opposite mode of vr3 splicing regulation between human and mouse (exon 12 and 13 by their numbering method). In addition, they found another novel exon just upstream of the vr3. The exon is alternatively spliced in human but not in mice, and suggested to contain another syntrophin binding motif, although the transcripts containing this exon are less expressed in skeletal muscle.

## 3. Structure of Dystrobrevin Protein and Localization in Striated Muscle

α-Dystrobrevin is comprised of four major domains, two EF-hand motifs that potentially bind calcium, a ZZ-domain, an α-helical coiled-coil domain containing a dystrophin binding site, and a tyrosine kinase substrate domain (Figure 1) [7]. α-Dystrobrevin 1, α-dystrobrevin 2 and α-dystrobrevin 3 bind to the sarcoglycan complex via the *N*-terminal region [8]. α-Dystrobrevin 1 and α-dystrobrevin 2 bind dystrophin through the highly conserved coiled-coil domain [2,7]. α-Dystrobrevin 1 and α-dystrobrevin 2 have syntrophin binding sites at the upstream of the coiled-coil domain [19].

α-Dystrobrevin 1, which has a unique *C*-terminal tyrosine kinase substrate domain [1,4], is localized in the sarcolemma and is abundant at the neuromuscular junction (NMJ), especially in the crest of the junctional folds [11,14,15,20]. α-Dystrobrevin 2 is localized around the entire circumference of the sarcolemmal membrane including the NMJ, and concentrated in the troughs of the folds [11]. α-Dystrobrevin 2 co-localizes with only dystrophin at the NMJ, while α-dystrobrevin 1 co-localizes with both dystrophin as well as utrophin which shares structural and functional similarities with dystrophin [11]. α-Dystrobrevin 3, which lacks both syntrophin and dystrophin binding sites, is capable of binding to the sarcoglycan complex via the *N*-terminal region [8]. α-Dystrobrevin 3 has been suggested to be localized in the cytoplasm [21].

## 4. The Role of Dystrobrevin for Structural Integrity of Muscle—Interaction with Cytoskeletal Binding Partners

**Figure 2.**
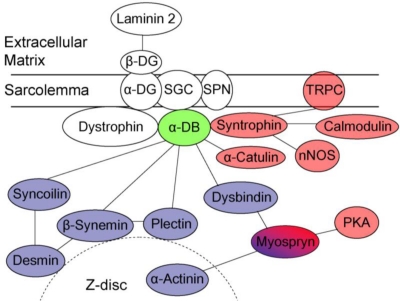
Schematic illustration of the dystrophin-glycoprotein complex network in muscle. α-Dystrobrevin (α-DB) forms a core part of the DGC with dystrophin, syntrophin, α-dystroglycan (α-DG), β-dystroglycan (β-DG), sarcoglycan complex (SGC), and sarcospan (SPN). Proteins involved in structural integrity and signaling are highlighted in purple and red, respectively.

α-Dystrobrevin is a component of DGC, which is indispensable for the structural integrity of muscle. As mentioned above, it directly associates with dystrophin at its coiled-coil domain and with sarcoglycan complex in its *N*-terminal half. As is the case with dystrophin, α-dystrobrevin may also function as a structural scaffold linking the DGC to the intracellular cytoskeleton. By using the yeast two-hybrid and co-immunoprecipitation analysis, several groups identified additional α-dystrobrevin-binding proteins: syncoilin, β-synemin, and dysbindin [22–24] (Figure 2). Syncoilin and β-synemin are both intermediate filament (IF) proteins. The IFs play a structural role by forming an important part of the cell cytoskeleton and providing mechanical stability to the cells [25]. In muscle cells, the IFs encircle the Z-lines of each integral myofibril, thereby connecting all adjacent myofibrils and linking the Z-lines of the peripheral layer of cellular myofibrils to the sarcolemma [26].

Syncoilin is highly expressed in striated muscle, and co-localizes with α-dystrobrevin at the NMJ and sarcolemma. Syncoilin interacts with α-dystrobrevin via vr3 and its flanking region [22]. Syncoilin also binds to desmin, a muscle-specific intermediate filament protein, and has been thought to organize desmin filaments to the Z-line [27]. The α-dystrobrevin/syncoilin interaction provides a further physical linkage between the DGC and the cytoskeleton, in addition to the well-characterized linkage between dystrophin and actin. Interestingly, syncoilin-null mice show a reduced force-generating capacity, suggesting that the link with syncoilin may be important for force transduction during contraction, although the DGC is still intact without syncoilin [28]. In addition, the increased syncoilin immunolabeling was reported in the sarcolemma of immature regenerating fibers in patients with Duchene muscular dystrophy [29] and mouse models of muscle disease [22]. This suggests that syncoilin and its binding partner, α-dystrobrevin +vr3, may be required for muscle regeneration in response to injury.

β-Synemin is a large, heteropolymeric IF protein that forms IFs with other major IF proteins, such as desmin [23]. β-Synemin interacts and co-localizes with α-dystrobrevin at the NMJ and myotendinous junction (MTJ) [23,30]. The α-dystrobrevin/β-synemin interaction provides another connection between the IF networks and the DGC. Furthermore, β-synemin interacts with plectin, a linker protein of IFs to Z-discs [31]. Interestingly, plectin is also suggested to interact directly with α-dystrobrevin. Taken together, these interactions between α-dystrobrevin and IF proteins would stabilize the sarcolemma and protect it against contraction-imposed stress by tethering IFs to the DGC at the sarcolemma (Figure 2).

Dysbindin is a ubiquitously expressed coiled-coil-containing protein, although the expression in muscle is relatively low [24]. In skeletal muscle, dysbindin is located at the sarcolemma. Dysbindin binds to α-dystrobrevin through coiled-coil domains and interact with myospryn [24,32]. Lastly, myospryn is a muscle-specific protein, localized to the sarcolemmal region of skeletal muscle. Myospryn is suggested to function as a docking platform for additional structural proteins and signaling molecules, such as α-actinin 2 and protein kinase A, respectively [33,34].

## 5. The Role of Dystrobrevin in Signaling—Interaction with Syntrophin

α-Dystrobrevin 1 and α-dystrobrevin 2 bind directly with syntrophin, which is a modular adaptor protein thought to be involved in signaling [35–38] (Figure 2). Skeletal muscle contains several isoforms—α-, β1-, β2-, and γ2-syntrophin—encoded by different genes [38–42]. α-Syntrophin is the major isoform in skeletal and cardiac muscles [36,38,41], being expressed in the sarcolemma and NMJ [14,20]. β1-Syntrophin also localizes at the sarcolemma and NMJ, whereas β2- and γ2-syntrophin are largely confined to the NMJ. With regard to the syntrophin binding site on Torpedo 87K protein, a homologue of human α-dystrobrevin 1, Dwyer *et al.* mapped the α-syntrophin binding site to a region analogous to exons 12–13 of human α-dystrobrevin [37], and Ahn *et al*. reported a β1-syntrophin binding site in a region corresponding to exons 13–14 of human α-dystrobrevin [36]. From yeast two-hybrid study, Newey *et al.* identified another binding site situated within the vr3 region in mouse, and suggested that α-dystrobrevin contains two independent syntrophin binding sites in tandem [43]. Recently, we directly confirmed that α-syntrophin interacts with α-dystrobrevin via the vr3 domain in human muscle and revealed dramatic decrease in binding between α-syntrophin and α-dystrobrevin mutant in vr3 [17]. α-Syntrophin knockout mice exhibit marked hypertrophy during muscle regeneration and deranged NMJ accompanied by impaired ability to exercise, suggesting a role of α-syntrophin in the regulation of muscle volume and formation of highly organized NMJ [44,45].

Syntrophin has the potential to co-ordinate the assembly of several important proteins such as neuronal nitric oxide synthase (nNOS) [46], stress-activated protein kinase-3 [47], Grb2 [48], and calmodulin [49,50] to the DGC. nNOS generates NO from L-arginine in many different cells. nNOS at the sarcolemma regulates local blood flow in contracting skeletal muscle in part by antagonizing sympathetic vasoconstriction [51]. The expression of nNOS is decreased in many muscular dystrophies, resulting in decreased vasodilation [52]. Consistent with nNOS being associated with α-syntrophin in muscle, α-syntrophin knockout mice lack sarcolemmal nNOS [53]. It is also reported that normalized production of NO protects dystrophin-deficient *mdx* muscle from degeneration [54]. Recently, transient receptor potential channels (TRPCs) were also found to associate with α-syntrophin [55]. These channels anchored to the DGC are suggested to form a signaling complex that modulates cation entry and regulates calcium homeostasis in skeletal muscles [56]. Calcium homeostasis and calcium-dependent signaling pathways play an important role in regulating muscle contractility, metabolism, and gene expression [57]. Thus, these signaling pathways via α-dystrobrevin/syntrophin may have a significant impact on muscle function.

Very recently, α-catulin, a catenin/vinculin-related molecule, was identified as a binding partner of α-dystrobrevin 1 [58]. α-Catulin, which co-localizes with α-dystrobrevin at nerve bundles and blood vessels, is thought to regulate α_1D_-adrenergic receptor signal transduction. It was shown that α-catulin interact with α-dystrobrevin 1 at its *C*-terminal domain. Since α-catulin is ubiquitously expressed, including in skeletal muscle [59], the interaction between α-dystrobrevin and α-catulin may be involved in some forms of receptor-mediated signaling in skeletal muscle.

## 6. Dystrobrevin in Muscle Disease

Despite the evidence that abnormalities of the DGC components cause various muscular dystrophies, so far no mutation in α-dystrobrevin has been reported in muscular dystrophy patients. However, a missense mutation was found in a four-generation Japanese family with left ventricular non-compaction (LVNC) [60], a cardiomyopathy, often associated with neuromuscular disorders [61]. LVNC is characterized by a pattern of prominent trabecular meshwork and deep inter-trabecular recesses, and is thought to be caused by arrest of normal endomyocardial morphogenesis [62].

Histological analysis showed that the level of α-dystrobrevin is greatly reduced at the sarcolemma of patients with DMD and SD-LGMD, suggesting that dystrophin and sarcoglycan are required for anchorage of α-dystrobrevin at the sarcolemma [21]. On the other hand, Jones *et al.* analyzed the expression of α-dystrobrevin by immunohistochemistry in biopsies from 162 patients with myopathies of unknown etiology with normal staining for dystrophin and other dystrophin-associated proteins [14]. Among the patients, deficiency of α-dystrobrevin was found in 16 patients, while no mutation was identified in the coding region of the α-dystrobrevin gene. Although there was a significant variety in severity and age of onset, all patients presented with congenital-onset hypotonia and weakness, indicating a new disease entity caused by α-dystrobrevin deficiency.

In addition, mice lacking α-dystrobrevin exhibit skeletal and cardiac myopathies, defects of NMJ maturation, and abnormal myotendinous junctions [9,63,64]. At NMJs in α-dystrobrevin knockout mice, the distribution of acetylcholine receptor (AChR) becomes granular in appearance with ragged edges. Myotube cultures from α-dystrobrevin knockout mice forms normal AChR clusters; however, these clusters quickly disappear into micro-aggregates, suggesting that α-dystrobrevin is not required for initial formation of the postsynaptic apparatus at the NMJ, but indispensable for its maturation and stabilization. Despite the presence of a structurally intact DGC in the sarcolemma, these mice show a striking displacement of nNOS from the sarcolemma and impaired NO-mediated signaling [9]. Moreover, the biochemical association between dystrophin and β-dystroglycan is impaired in α-dystrobrevin knockout mice, suggesting a role for α-dystrobrevin in stabilizing the DGC [65].

Furthermore, we revealed mis-regulated alternative splicing of α-dystrobrevin in muscle from myotonic dystrophy type 1 (DM1) patients [17]. DM1 is proposed as a “spliceopathy”, *i.e*., a *trans*-effect on the alternative splicing of many RNAs, leading to inappropriate expression of aberrantly spliced products, such as Cl channel, ryanodine receptor and dystrophin [66–69]. α-Dystrobrevin mRNA including vr3 is increased in both skeletal and cardiac muscle of DM1 patients. Interestingly, the splicing abnormality is correlated with the muscular disability of DM1. The aberrantly spliced α-dystrobrevin isoform has an increased binding capacity for α-syntrophin, thereby recruiting excess α-syntrophin to the sarcolemma in DM1 muscle.

## 7. Concluding Remarks

A number of proteins that comprise the link between striated muscle Z-disc and peripheral structures, such as the costamere, have been discovered. Recent studies suggest that this protein network acts as a structural and signaling center for striated muscle to ensure coordinated contractile activity. The importance of the network to normal muscle function has emerged from investigations into the causes of muscular dystrophies and cardiomyopathies. One of the key players in the network is α-dystrobrevin that mediates signaling and structural function of the DGC. Although functional differences among the various splicing variants and detailed roles of their counterparts still remain unknown, future studies will shed much more light on the importance of α-dystrobrevin in striated muscle and implications for muscular dystrophy.
